# Forecasting the future? Differential allocation of maternal hormones under different social contexts in the blue tit *Cyanistes caeruleus*

**DOI:** 10.1098/rsos.250150

**Published:** 2025-04-02

**Authors:** Alejandro García Antón, Wendt Müller, Jorge García-Campa, José Javier Cuervo, Lucía Mayor-Fidalgo, Nazaret Cubas, Jimena Lopez-Arrabe, Judith Morales

**Affiliations:** ^1^ Universiteit Antwerpen, Antwerpen, Belgium; ^2^ National Museum of Natural Sciences, Madrid, Spain; ^3^ Department of biology, Universiteit Antwerpen, Antwerpen, Belgium; ^4^ Department of Evolutionary Ecology, National Museum of Natural Sciences, Madrid, Spain; ^5^ CIBIO-InBIO, Universidade do Porto, Vairão, Portugal; ^6^ Department of Physiology, Complutense University of Madrid Faculty of Veterinary, Madrid, Spain

**Keywords:** testosterone, androgens, developmental programming, prenatal environment, social networks, phenotypic plasticity

## Abstract

Sociability, i.e. the tendency to interact with other individuals, varies significantly within populations, with some individuals being consistently more sociable than others. Variation may be maintained because the balance between costs (e.g. increase in aggressive disputes, infection risk) and benefits (e.g. information exchange, cooperation) of sociability varies with the environmental context. At the proximate level, apart from genes, mothers transfer non-genetic compounds to their offspring that can influence the development of social skills. In this context, they may adjust their offspring’s sociability to match the social environment they will experience after birth, for example, via prenatal hormones. To test this, we experimentally manipulated the social density as perceived by blue tit females before egg laying. We subsequently measured yolk testosterone concentrations and social interactions among family members post-hatching. Females that were exposed to a simulated high social density transferred less testosterone to their eggs than control females. Network average degree (i.e. the number of social interactions of the brood) was not affected by the social density treatment, but broods with lower yolk testosterone concentrations showed a higher network average degree. This suggests that mothers experiencing an environment with high social density (but not increased resource competition) deposit less yolk testosterone to produce offspring that are probably less aggressive but more sociable.

## 1. Introduction

Sociability is the tendency to interact with other individuals without aggressive or reproduction-related purposes [1]. It is a fundamental trait with significant effects on fitness because social interactions facilitate information exchange [2–4], which is vital for the resolution of conflicts [5,6] and cooperation [7,8]. Social information can also lead to better access to resources (e.g. [9–15]) or enhanced protection against predators [16–18]. Yet, having a high number of social interactions may also entail costs, such as an increased likelihood of becoming infected with contagious diseases [9,19–21], experiencing aggression [22,23] or facing competition [24,25]. Thus, sociability probably has broad ecological consequences, with the balance between the costs and benefits of sociability varying according to the environmental context, such as social density [26–31].

Sociability represents one of the five major axes of animal personality, and like other personality traits, there is significant variation among individuals in their sociability [1]. Such among-individual variation may partly arise early in life, when the phenotype is highly sensitive to environmental inputs [32,33]. For example, nestling gulls reared in more socially dense areas of the colony show a higher number of social contacts [34]. Similarly, in zebra finches, individuals raised in larger broods occupy more central positions in their social network after fledging [35].

Intriguingly, previous studies have shown that even the prenatal social context influences the development of the social phenotype. Offspring born to mothers who experienced high social density were more socially interactive and less aggressive [36,37]. From a proximate perspective, such early environmental effects could arise through different pathways. Offspring can directly perceive environmental cues during the embryonic stage and modify their behaviour accordingly [38–41]. Alternatively, offspring can indirectly respond to cues provided by the parents, such as acoustic signals during embryo development (reviewed by [42]; see also [41]) or specific compounds (e.g. hormones) transferred by their mothers, as these may contain information about the environment [43–45].

Such maternal effects have been suggested as an adaptive mechanism by which mothers communicate information to their offspring about the prenatal environment to better prepare them for living in that environment. This concept has been defined as ‘anticipatory maternal effects’ [46,47]. In birds, this topic has been intensively studied. For instance, research has shown that the concentrations of maternally derived yolk testosterone vary with the social environment experienced by the mothers. Higher social densities usually lead to higher maternal yolk testosterone levels, as demonstrated in several experimental studies (house sparrows [48]; European starlings [49]; tree swallows [50]). This positive relationship has also been found in a number of correlational studies across different species (house sparrows [51]; American coots [52]; European starlings [53]; great tits [54]; Eastern bluebirds [55]). However, negative correlations (black-headed gulls [56]; barn swallows [57]) or no effect of social density on yolk androgen levels (collared flycatchers, [58]) have also been reported. This variability might be due to the fact that a high social density could limit resource availability, leading to increased competition for resources [59]. Indeed, it has been shown that food availability before egg laying can affect the deposition of androgens in the yolk [60–63]. Yet, to our knowledge, no previous experimental study has attempted to separate the effects of social density and resource availability in the offspring’s prenatal environment. A good approach to achieve this is to manipulate only the perception of social density experienced by mothers prior to egg laying, rather than the actual social density, i.e. the actual number of individuals competing for those resources.

The observed effects of the maternal social environment on the deposition of yolk testosterone could indeed facilitate the adjustment of the offspring to the postnatal social environment, because maternal yolk testosterone has both short-term effects on offspring behavioural traits, like begging to the parents and competitive interactions among siblings, as well as long-term effects on social dominance and dispersal (reviewed by [64,65]). However, little is known about the effects of yolk testosterone on offspring sociability. This is intriguing, since how well individuals can deal with the social environment (which, in fact, shaped maternal yolk testosterone deposition in previous studies) may hinge on their sociability.

In this study, we investigated whether the social component of the maternal environment modulates yolk testosterone deposition into the eggs and whether such early maternal effects influence offspring social behaviour early in life. For this purpose, prior to and during egg laying, we exposed part of our study population of blue tits (*Cyanistes caeruleus*) to playbacks of conspecifics, simulating a high social density without changing the actual social density. The other part of the population was exposed to a control treatment. We then collected one egg per clutch to analyse the maternal yolk testosterone concentrations. After hatching, we measured offspring behaviour, including the social contacts among siblings in the nest, and applied a social network approach following previous studies in the blue tit and in the closely related great tit (*Parus major*) (see [11,66]). As the parents are also part of the offspring’s early social environment, we also explored how the nestlings interact with their parents via begging. Finally, we measured nestling body mass to analyse possible functional consequences of the social behaviour of the nestlings.

We expected higher yolk testosterone concentrations in the clutches laid by females exposed to perceived high social density and predicted that offspring born from eggs laid by females exposed to the high social density treatment would be more sociable. Specifically, we expected them to show a higher social network average degree, which is a measure of contact interactions in the brood [11].

## 2. Methods

### 2.1. General methods and study species

The study was carried out in Miraflores de la Sierra, Madrid, central Spain (40° 48′ N, 03° 47′ W) during the spring of 2021. We investigated a wild blue tit population breeding in nest-boxes located in a deciduous forest primarily dominated by Pyrenean oak (*Quercus pyrenaica*) at an elevation of 1250 m. The blue tit is a socially monogamous species with intense bi-parental care after hatching. Clutch size is relatively large in our study population (on average 9.6 eggs ± 1.8 s.d.; *n* = 464 clutches; range 4−14), resulting in numerous contact interactions among siblings in the nest. This makes the blue tit an appropriate model to analyse offspring social network structure (see [66]). Additionally, oviparous animals such as blue tits are excellent model systems to study early maternal effects, as mothers transfer all the necessary material for offspring development into a self-contained package: the egg [67].

The experimental manipulation (see §2.2) took place from mid-March to the beginning of May, i.e. until the onset of incubation in the last nest included in the experiment. Nest-boxes were visited every 2 days to monitor the beginning of nest construction and laying date (first egg laid). Once laying started, the first and second eggs of each clutch were marked to identify the third-laid egg for collection. Nests were visited daily to collect the third egg of each clutch on the day it was laid, to record clutch size and to determine the onset of incubation. The third egg was weighed in the field to the nearest 0.01 g and replaced with a fake egg of similar size, colour and weight to prevent females from rejecting it. Collected eggs were kept cool during transport and stored at −80°C on the same day of collection until analysis (see §2.3). Additionally, we recorded the hatching date, with day 0 denoting the hatching of the first nestling.

In the second week after hatching (days 9−12), we captured both adult blue tits when they entered the nest-box to feed the nestlings. Adults were weighed using a digital scale to the nearest 0.01 g. Their sex was determined visually based on plumage characteristics and the presence of an incubation patch. The first adult captured in each nest was marked by bleaching part of the back feathers using a commercial bleach (L’Oréal Paris Colorista). This allowed us to distinguish between male and female parents during subsequent video observations. This method has been used in previous experiments, and no effects on adult behaviour were detected (e.g. [68]). On day 12, nestlings were ringed, weighed with a digital scale to the nearest 0.01 g and marked individually on the head or wings with a white permanent marker (Edding 751). We also collected a blood sample from the brachial vein of each nestling for molecular sexing (see §2.5).

### 2.2. Experimental manipulation of social density

The study area (approx. 52 hectares) was divided into two large and two small plots (see a map of the study area in the electronic supplementary material). One large and one small plot were assigned to the high social density group, containing 55 and 28 nest-boxes, respectively; similarly, one large and one small plot were assigned to the control group, containing 69 and 18 nest-boxes, respectively. Thus, the (simulated) high-density and control treatments included, in total, 83 and 87 nest-boxes, respectively, spread across similarly sized areas. Occupancy rates of nest-boxes in previous years (2017−2019) did not differ significantly between the two treatments (mean ± s.d.; high-density plots: 54.6 ± 6.8%; control plots: 55.5 ± 5.5%). We expect the nest-box occupancy rate to be a reliable representation of the actual social density in the area since the forest is relatively young and there are not many natural cavities available. Each plot was separated by at least 77 m from the others to minimize the possibility that individuals could hear the playbacks from another plot.

From mid-March (16 March) onwards, almost three weeks prior to the laying of the first egg (4 April in the study year), we exposed blue tit adults daily to either recordings simulating high social density or to recordings of a control species. This treatment continued until the 3 May, when most pairs had completed their clutch (except for two nests, each with a different treatment). A total of 38 speakers (19 per treatment) were distributed across the study area: six in each of the two small plots and 13 in each of the two large plots. The positions of each speaker were fixed throughout the experiment and nest-box were registered with a GPS device, and the distances between them were calculated using the function *distGeo* from the package *geosphere* [69] in R [70]. The range of distances to the closest speaker in the whole sample of nest-boxes was 10.34−74.75 m. The average distance of nest-boxes to the nearest speaker was 23.8 and 35.5 m for the two control plots and 26.7 and 29.4 m for the two high-density plots. Each speaker (speaker model: Mifa A1 waterproof and wireless, China) was programmed to play a playlist of 8 h, starting from around 9.00 and ending around 17.00. From the second week of April, the playlists were extended to 10 h (from around 9.00 to 19.00), given that daylight hours increase as the season progresses. During the playlists, vocalizations were interspersed with silent periods, especially more frequent during midday hours (12.00 to 16.00). This pattern aimed to replicate natural conditions, as blue tits usually reduce singing activity during midday hours [71]. Overall, vocalizations were broadcast for 25% of the total playlist duration.

To simulate a high social density environment for the blue tits, vocalizations of male songs and female calls (excluding alarm calls) were broadcasted. For the control plots, vocalizations of male and female common chaffinches (*Fringilla coelebs*) were broadcasted. Common chaffinches are common in the study area, but do not breed in nest-boxes, have different feeding habits and are not expected to influence the social environment of blue tits. All recordings were obtained from Xeno-Canto database (https://xeno-canto.org/). For both treatments, we aimed to include only vocal repertoires from the Iberian Peninsula to prevent potential abnormal responses to vocalizations from distant, vocally dissimilar populations. However, this was not possible for female vocalizations of either species, so we included some from other European populations. For each treatment, we created two playback lists containing vocalizations from 10 different individuals per list, which were randomly allocated for the final broadcast. Every morning, we replaced the batteries in the speakers, and to prevent habituation effects, we alternated between the two playbacks lists (within the same treatment) for each speaker. Similarly, we alternated the order (clockwise or counter-clockwise) in which the speakers were activated daily to avoid potential biases related to start times.

Finally, we conducted standardized censuses in the morning to quantify the social behaviours of the territory-holding male and female blue tits (see [72] for a similar experimental set-up). Over a period of 42 days, we performed four censuses each morning at a distance of approximately 10 m from four different speakers (two per treatment in two different plots) to measure the response to either blue tit or common chaffinch playbacks. On the first day, we randomly selected the speakers, and on subsequent days, we switched to different speakers from the corresponding treatment, ensuring an equal proportion of censuses per treatment and that all speakers were included a similar number of times. Before starting the playbacks, we conducted a census to record the presence of blue tits or chaffinches within 10 m of the speaker for 1 min, either by sight or by sound. Then, we activated the speaker, and 5 min later, we initiated a second census. We recorded the responses of blue tits or chaffinches to the playback (0 = no response; 1 = at least one individual detected within 10 m of the speaker). The analysis employed a mixed model with a binomial distribution, using the presence/absence of blue tits as the dependent variable, speaker ID as a random factor, treatment and observer ID as fixed factors, and the number of blue tits observed before the playback (maximum of two individuals) as a covariate. This ensured that the number of individuals present before the census was controlled for, but it did not affect the results (see below). We did not include the sex of the birds, as it was impossible to distinguish sexes from such large distances. The playback might have been perceived as a territorial intrusion, for example, when in close proximity to the nest-box. However, territorial intrusions are known to increase with rising social densities, so it is likely that both exert similar effects.

### 2.3. Yolk hormone analysis

This analysis was performed at the Ecophysiology Laboratory of the National Museum of Natural Sciences–Spanish National Research Council (MNCN-CSIC). The third egg of each clutch was collected, kept cool during transport and stored at −80°C on the same day of collection until analysis. Yolk hormone extraction was performed following [73] with some modifications. On the day of hormone analysis, each egg was slowly defrosted to isolate the yolk, which was weighed with a high-precision scale to the nearest 0.001 g. Subsequently, each yolk was diluted (1 : 2 w/v) in double-distilled water. We took half of the sample and extracted steroids by adding 3 ml of a mixture of petroleum ether and diethyl ether (30 : 70) to the diluted yolk sample. The mixture was vortexed for 10 min and then centrifuged for 10 min at 4°C and 1500 r.p.m. The ether phase was decanted after snap-freezing the tube in an ethanol bath with dry ice. This procedure was repeated a second time. Both ether phases were combined in a single tube and evaporated to dryness in a heating block at 50°C under a stream of nitrogen. To remove protein residues, the extract was resuspended in 0.5 ml of 90% ethanol, vigorously mixed, and kept at −20°C overnight. The next day, the extract was centrifuged for 10 min at 4°C and 1500 r.p.m., and the supernatant was transferred to a new clean tube. Subsequently, it was dried again in a heating block at 50°C under a stream of nitrogen and resuspended in 0.5 ml of steroid-free serum (DRG Labs, Germany). After a 10 min vortexing, the samples were transferred to Eppendorf tubes and frozen at −20°C until analysis.

To measure testosterone concentrations, we used an enzyme immunoassay according to the manufacturer’s protocol (Testosterone ELISA; ref. EIA−1559; DRG, Germany). The cross-reactivity of these kits is 0.8% between testosterone and 5α-dihydrotestosterone and 0.9% between testosterone and androstenedione. Inter-assay variability was assessed using the coefficient of variation (CV) of known controls across four assays (CV = 5.20%, *n* = 28). All samples were run in duplicate in each assay to assess intra-assay variability (CV = 6.89%, *n* = 143). A Synergy HT Multi-Mode Microplate Reader (Biotek, USA) at 450 nm was used to measure changes in absorbance according to the EIA-1559 procedure.

### 2.4. Behavioural observations

On day 12, we transferred the nest from the original nest-box to a recording nest-box, which was placed in exactly the same position and orientation as the original. The recording box is a similar-sized nest-box with an opening in the ceiling that is covered by a black box in which we initially placed a fake camera. This set-up has been successfully used in previous studies (e.g. [66,68,74]). We could not place recording nest-boxes in the area for the whole breeding season as they do not provide protection against predators such as woodpeckers. The next day, day 13, we placed a night-vision video camera (wide-angle 8-LED IR DVR camcorder, DX, China) on the recording nest-box, approximately 10 cm above the nest [66]. We recorded the nest for 90 min, excluding the initial 30 min and the final 10 min of the video to mitigate potential interference from researchers during camera placement and removal. Then, we registered the behaviour of all family members for the first 30 min. We previously validated that feeding rates registered over 30 min strongly correlate with those observed over 1 h (Pearson’s *r* = 0.84, *p* < 0.001, *n* = 45, data from [75]). A single observer analysed all video recordings and was unaware of the social density manipulation. All videos were recorded at the same age for all nests (day 13), when no speakers were active any more.

For each feeding event, we recorded whether the adult was marked or not and assessed the nestlings’ begging intensity. Begging intensity was rated on a 5-point scale following [76]: 0 = calm, 1 = weak gaping, 2 = gaping and neck stretched, 3 = gaping with neck stretched and standing, and 4 = gaping with neck stretched, standing and wing flapping. For each nestling, we calculated the average overall begging intensity across all feeding events from both parents combined. Additionally, we obtained sex-specific scores by averaging begging intensities for each parent separately. For each parent, we also recorded the total number of feeding events and the average time spent in the nest-box per visit.

From the 30 min recorded in the videos, we scored positions within the nest and derived the social metrics from 10 successive events occurring between parental visits, the so-called ‘parent-absent events’, when no parent was present. In three out of the 42 nests analysed, the 10 events had to be reduced to eight or nine since the parents did not feed frequently enough. We considered that a parent-absent event started at least 5 s after the parents had left the nest and once all chicks had become calm and lasted until the parents returned. We excluded feeding events due to the typically intense vocalizations and postural begging behaviour exhibited by nestlings in the presence of their parents, which would complicate the identification of individual positions. Nestlings change positions mainly during or soon after feeding events (personal observation). Therefore, we recorded nestling positions at 10 single points in time immediately after the start of each parent-absent event, pausing the video a minimum of 5 s after the parents had left the nest. Although the nest cup is a relatively restricted space, nestlings are free to change their position, either occupying more central or more peripheral positions in the nest cup. Many factors could motivate the nestlings to be in physical contact with their nestmates (e.g. being closer to favourable feeding spots or regulating body temperature). However, a previous study found that blue tit nestlings signalling lower conditions (experimentally reduced UV chroma) occupy more peripheral positions soon after manipulation [66]. This implies that movements are not random and that they inevitably affect the level of social interactions among siblings.

Following [11], we defined a social interaction as direct physical contact between two nestlings. With this information, we created an *n* × *n* matrix, where *n* represents the number of individuals in the nest, and each cell indicates the presence (1) or absence (0) of an interaction between two individuals. Then, we extracted the number of interactions of each nestling in each of the 10 parent-absent events analysed and averaged these values to have a single score representing the average number of interactions of each individual, the *unweighted individual degree*. As a measure of how frequently individuals in the group associated with each other, we calculated *unweighted network average degree* [77], which represents the average number of physical interactions among all nestlings within a nest (see also [66]). Similar social network analyses have been effectively employed in previous studies [11,66], demonstrating their utility in examining how social network structure influences parental feeding behaviour and nestling recruitment (fitness) [11]. Additionally, we elaborated a weighted matrix averaging the values of all the binary matrices (on average, 10). In this matrix, the values in the cells indicate the proportion of time two individuals were associated. We did this to calculate the *weighted individual degree* and then the *weighted network average degree*, facilitating comparisons with previous studies (e.g. [11]).

### 2.5. Molecular sexing of nestlings

This analysis was performed at the Laboratory of Molecular Systematics and Population Genetics of the National Museum of Natural Sciences–CSIC. DNA was extracted from blood samples using the Qiagen DNeasy Blood and Tissue kit (Qiagen Inc., Valencia, CA, USA). Sex identification was performed by polymerase chain reaction (PCR) amplification of the CHD-W and CHD-Z genes with primers P2 and P8, following [78] with slight modifications [74]. The sex of two chicks from different nests could not be determined due to unsuccessful DNA extraction, and they were excluded from analyses involving nestling sex.

### 2.6. Statistical analyses and sample size

The statistical analyses were conducted in the R statistical environment (v. 4.3.1. [70]). Models were visually inspected using the *performance* package [79]. Multi-collinearity was assessed using the variance inflation factor (VIF), and no problematic variables were detected (all VIF values less than 5).

First, we used linear models (LMs) to assess the effect of treatment (high social density vs control) on yolk testosterone concentration. The independent variables included treatment, laying date, clutch size and distance to the nearest speaker. Additionally, we checked the effect of our treatment on egg mass and clutch size to test whether treatment had an effect on settlement decisions and, hence, on the quality of the adults. The independent variables included in the egg mass model were treatment, laying date, clutch size and distance to the nearest speaker. In the clutch size model, the independent variables were treatment, laying date and distance to the nearest speaker. For the analysis of unweighted network average degree, the model included treatment, yolk testosterone concentration, recording date and brood size. In addition, we ran the same model but with weighted network average degree as the dependent variable. The results of this analysis can be found in the electronic supplementary material.

Second, we conducted linear mixed models (LMMs) using the *lme4* package [80], with nest identity as a random factor, to examine the effects of treatment and yolk testosterone on individual nestling parameters (body mass and begging) and on parental behaviour (number of parental feedings and time spent in the nest). For nestling body mass (at the individual level), our independent variables included treatment, yolk testosterone concentration (at the clutch level), ringing date, brood size and nestling sex. In the analysis of begging intensity (also at the individual level), we included the same covariates as in the previous model, along with individual nestling body mass, parental sex and the interaction between parental sex and treatment.

For the number of feedings per brood, we included treatment, yolk testosterone concentration, recording date, brood size, parental sex and mean begging intensity of the brood as independent variables. Since mothers spent much more time inside the nest compared with fathers (mean ± s.d.; 28.0 ± 21.0 s vs 11.3 ± 2.71 s, respectively), we only analysed the effect of treatment on females. Thus, in this model, we excluded parental sex but added the number of feeding events as an independent variable.

We applied the *Anova* function from the *car* package [81] to perform type III *F*-tests for all models. In addition, for mixed models, we used *F*-tests with Kenward–Roger approximation to calculate degrees of freedom.

Initially, 84 nests were included in the experiment, but after removing the females that did not complete their clutch, 65 nests remained for hormone analyses (clutch size range: 6−12, average: 9.48). There was total brood failure in 12 nests, so 53 nests (417 nestlings) were available for the analyses of body mass (brood size range: 4−11, average: 8.45). Thirteen additional nests were excluded from behavioural analyses, either because of low recording quality or because the brood size was too small (less than four nestlings). Thus, there were 40 nests for the analyses of parental effort (i.e. feeding rate and time spent in the nest), with 39 females and 35 males, and 329 nestlings for the analyses of begging behaviour (brood size range: 5−11, average: 8.24), of which two nestlings could not be sexed. Finally, for network average degree, three additional nests were excluded because more than two nestlings could not be seen when analysing the parent-absent events. As a result, there were 37 nests available for network average degree analysis, encompassing a total of 304 nestlings (brood size range: 5−11, average: 8.22).

## 3. Results

### 3.1. Reaction to speakers

Our analysis confirmed that blue tits were significantly more likely to approach the speakers in the high-density treatment (broadcasting blue tit vocalizations) than in the control treatment (estimate ± s.e. for high density = 1.77 ± 0.41; *F*_1,151_ = 18.30; *p* < 0.001). The number of blue tits observed before the speaker was turned on had no effect (estimate ± s.e. = 0.002 ± 0.384, *F*_1,151_ = 0.00; *p* = 1.00). Similarly, common chaffinches were significantly more likely to approach the speakers in the control treatment (broadcasting chaffinch vocalizations) than in the high-density treatment (estimate ± s.e. for high-density treatment = −1.91 ± 0.54; *F*_1,151_ = 12.36; *p* < 0.001; same mixed model as before).

### 3.2. Prenatal maternal effects

**Figure 1.**
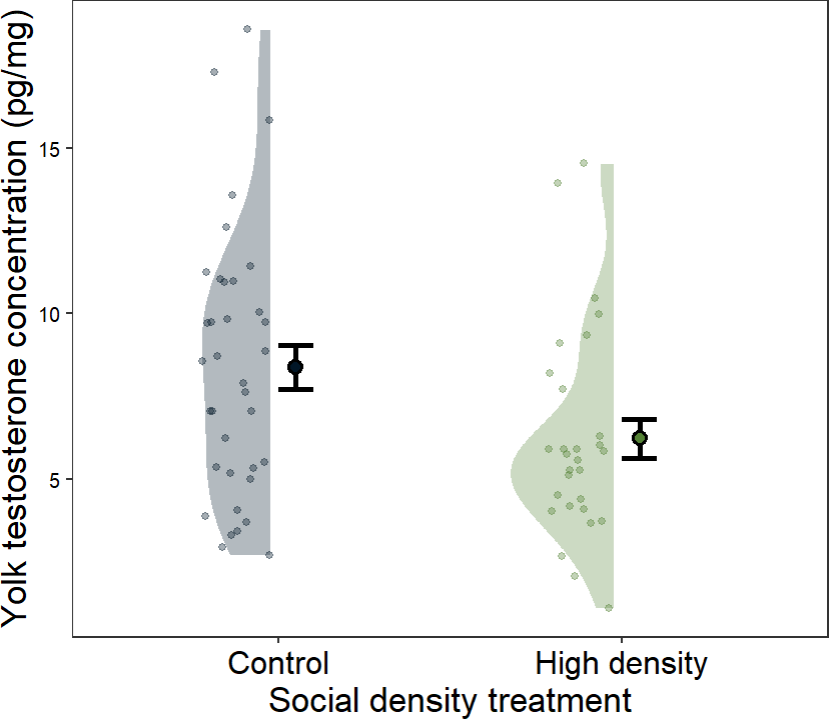
Effects of the social density treatment on yolk testosterone concentration. Means and standard errors are shown. Raw data points are plotted. Black and green areas represent the density distribution of values for control and high social density nests, respectively.

We found a significant effect of the experimental manipulation on the concentration of yolk testosterone (table 1). Females that perceived a high social density laid eggs with lower testosterone concentrations than control mothers (figure 1). Clutch size was not affected by the treatment (estimate ± s.e. = −0.46 ± 0.36, *F*_1_ = 1.61, *p* = 0.21). However, there was a positive and significant effect of the distance to the closest speaker on clutch size (estimate ± s.e. = 0.03 ± 0.01, *F*_1_ = 5.95, *p* = 0.02), with clutches having one additional egg for every 33.3 m of distance from the nearest speaker.

**Table 1.** Linear model showing the effect of the social density treatment on yolk testosterone concentration (pg mg^−1^). The *p*-values in bold indicate statistical significance (*p* < 0.05).

	estimate (s.e.)	*F_1_*	*p*
intercept	5.58 (3.33)	2.81	0.099
treatment (high density)	−2.06 (0.94)	4.79	**0.033**
laying date	0.04 (0.09)	0.15	0.70
minimum distance to speaker	−0.02 (0.03)	0.30	0.59
clutch size	0.31 (0.33)	0.87	0.36

### 3.3. Offspring network average degree

**Figure 2.**
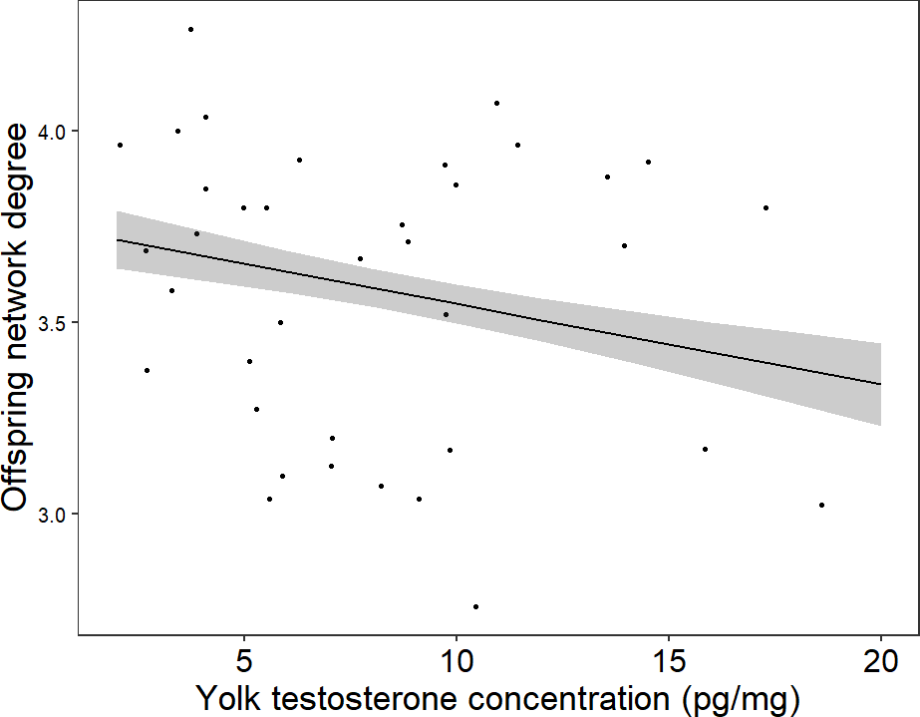
Offspring social network average degree in relation to yolk testosterone concentration. Raw data points are plotted. The regression line and shaded areas represent the predictions and standard errors of the model while holding brood size and laying date (the other two covariates) constant.

Nests with lower yolk testosterone concentrations had a higher network average degree (table 2; figure 2). As expected, brood size (i.e. number of interacting individuals) had a strong positive effect on network average degree (table 2).

**Table 2.** Effects of the social density treatment and testosterone concentration on network average degree (linear model), and on nestling body mass and begging intensity (linear mixed models with nest identity as a random factor). The *p*-values in bold indicate statistical significance (*p* < 0.05). The degrees of freedom for the numerator are 1 for all the linear mixed models.

	network average degree			nestling body mass			begging intensity		
	estimate (s.e.)	*F*_1_	*P*	estimate (s.e.)	*F*_df den_	*P*	estimate (s.e)	*F*_df den_	*P*
intercept	3.47 (0.56)	38.89	**< 0.001**	3.17 (2.09)	2.29_48.38_	0.14	1.76 (1.02)	3.00_36.13_	0.09
treatment (high density)	−0.01 (0.07)	0.02	0.89	−0.30 (0.34)	0.77_47.97_	0.38	−0.09 (0.15)	0.34_40.49_	0.57
yolk testosterone (pg mg^−1^)	−0.02 (0.01)	5.99	**0.020**	−0.03 (0.04)	0.43_47.94_	0.51	0.01 (0.02)	0.67_34.89_	0.42
nestling sex (male)	—	—	—	0.47 (0.04)	131.60_642.48_	**< 0.001**	0.02 (0.05)	0.10_301.78_	0.76
nestling body mass	**—**	—	—	—	—	—	−0.08 (0.04)	4.05_202.16_	**0.046**
parental sex (male)	**—**	—	—	—	—	—	0.10 (0.05)	4.08_286.55_	**0.044**
brood size	0.16 (0.02)	53.34	**< 0.001**	0.01 (0.09)	0.02_48.42_	0.90	0.01 (0.04)	0.06_34.54_	0.80
recording date	−0.02 (0.01)	4.99	**0.033**	—	—	—	−0.01 (0.02)	0.05_37.02_	0.82
ringing date	—	—	—	0.13 (0.04)	12.33_48.14_	**< 0.001**	—	—	—
treatment : parental sex	—	—	—	—	—	—	0.18 (0.07)	5.78_286.26_	**0.017**

### 3.4. Offspring begging and body mass

**Figure 3.**
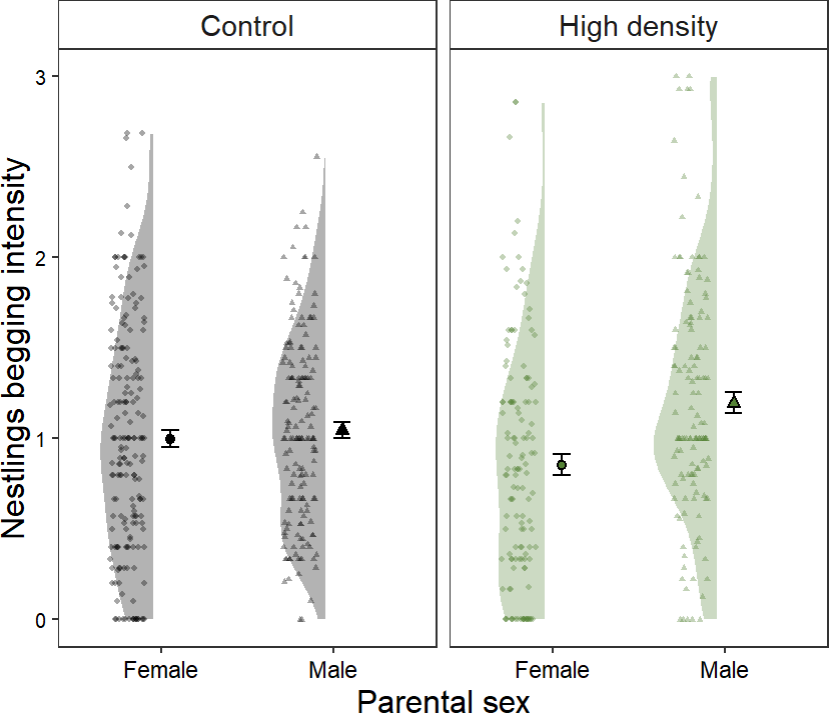
Offspring begging intensity in relation to the sex of the parent the social density treatment. Means and standard errors are shown. Raw data points are plotted. Circles and triangles represent the values for female and male parents, respectively. Black and green areas represent the density distribution of values for control and high social density nests, respectively.

There was no significant effect of treatment or yolk testosterone concentrations on begging intensity. However, we found a significant interaction between treatment and parental sex (table 2). Although nestlings generally beg with a higher intensity from the father, this difference was stronger in nests from the treatment group (figure 3). In addition, there was no significant effect of treatment on nestling body mass (table 2). Similarly, yolk testosterone concentrations did not significantly affect nestling body mass (table 2).

### 3.5. Parental care

**Figure 4.**
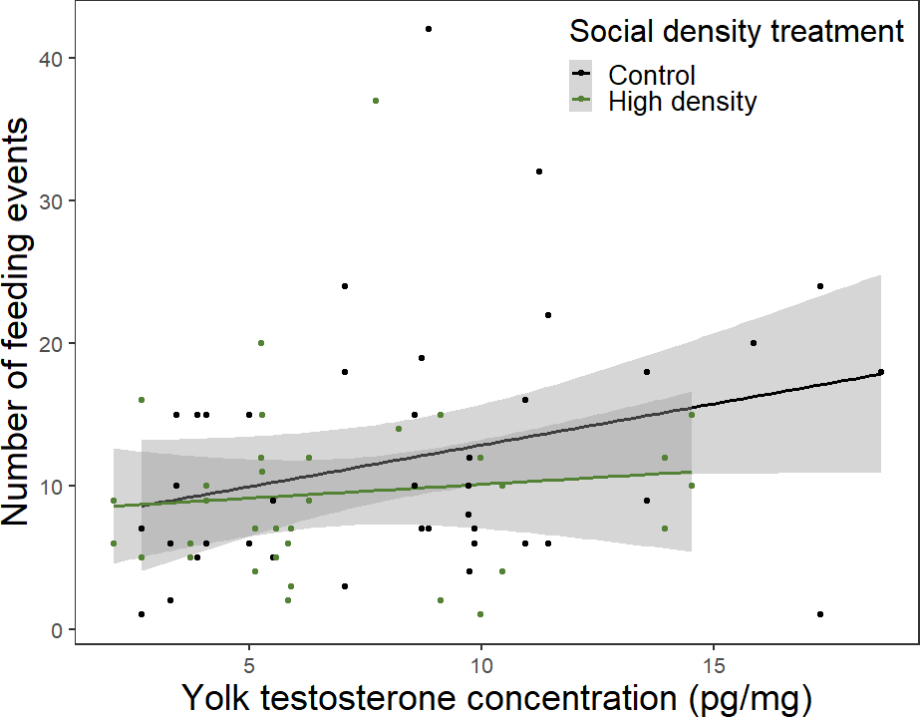
Number of feeding events provided by both parents in relation to testosterone concentration in the eggs and the social density treatment. Raw data points are plotted. Shaded areas represent the standard errors around the lines.

There was no significant effect of treatment on the number of feeding events (table 3). However, there was a non-significant but positive relationship between yolk testosterone and the number of feeding events (table 3; figure 4), with parents feeding their nestlings more often when yolk testosterone concentration was higher. There was also a non-significant trend suggesting that yolk testosterone had a negative effect on the time females spent in the nest (table 3). Additionally, females with larger broods spent less time in the nest (table 3).

**Table 3.** Effects of the social density treatment and testosterone concentration on the number of feeding events (linear mixed model) and the time spent in the nest by females (linear models). The *p*-values in bold indicate statistical significance (*p* < 0.05). The degrees of freedom for the numerator are 1 for all the linear mixed models.

	number of feeding events			female mean time in nest		
	**e**stimate (**s.e.**)	*F*_df den_	*P*	**e**stimate (**s.e.**)	*F*_1_	*p*
intercept	23.01 (13.09)	3.08_32.15_	0.089	66.20 (42.54)	2.42	0.13
treatment (high density)	−1.10 (1.83)	0.36_32.23_	0.55	7.11 (5.98)	1.41	0.24
yolk testosterone (pg mg^−1^)	0.41 (0.23)	3.23_35.74_	0.081	−1.45 (0.73)	3.90	0.057
parent sex (male)	1.73 (1.75)	0.97_37.06_	0.33	—	—	—
mean begging	0.10 (2.38)	0.002_42.79_	0.97	5.93 (8.32)	0.51	0.48
brood size	0.60 (0.55)	1.17_31.06_	0.29	−4.89 (1.81)	7.29	**0.011**
recording date	−0.39 (0.21)	3.66_33.51_	0.064	0.17 (0.66)	0.065	0.80
feedings	—	—	—	−0.43 (0.40)	1.12	0.30

## 4. Discussion

In this study, we experimentally tested whether a simulated high social density before and during egg laying modulates the allocation of maternal yolk testosterone by female blue tits, and whether this induces differential sociability [57]. Contrary to our predictions, we found that eggs in the simulated high social density treatment had lower yolk testosterone concentrations. Furthermore, we observed a negative effect of yolk testosterone on sociability, represented here by social network average degree, a measure of social contacts. The implications of these findings are discussed below.

### 4.1. Maternal yolk hormones

Blue tit females transferred less testosterone to their eggs when they were acoustically exposed to a simulated high social density, contrary to our prediction and the majority of previous studies on this topic (reviewed in [82–84]; but see [57]). However, in our experiment, we manipulated only the social cues in the females’ environment and not the actual social density, unlike earlier studies. With this approach, we aimed to avoid confounding effects of higher resource competition, which is typically intertwined with high social density, particularly when social density is experimentally enhanced. From a proximate perspective, higher levels of agonistic interactions among females breeding in high social densities could trigger the deposition of high levels of yolk testosterone, which may explain the mostly positive relationships observed in previous studies. This could be adaptive, as high yolk testosterone is typically associated with greater aggressiveness [85–87] and competitiveness in offspring (see meta-analysis in [84]), which could be beneficial if the offspring’s environment is characterized by high resource competition.

In our study, the observed negative effect of simulated high social density could possibly result from the perception of high social density without elevated levels of competition. Depositing lower concentrations of yolk testosterone by experimental females might be related to the priming of a more sociable phenotype in the offspring, which could be advantageous in environments where social ties and information exchange are critical for survival [88]. If sociability becomes more important than competitiveness, a reduced maternal allocation of yolk testosterone could be beneficial, resulting in less aggressive offspring who avoid costly aggressive interactions [22,23,25,89–93] while favouring non-aggressive encounters and the establishment of social bonds. Indeed, studies in both vertebrate and invertebrate species have found that more sociable individuals are, on average, less aggressive [94,95], which may facilitate group living and, thus, access to social information [3], food resources [2] and reduced predation risk [16–18]. However, less aggressive individuals might perform worse at territory defence and mate acquisition [96,97]. It remains to be determined whether the benefits of increased sociability outweigh the costs of reduced aggressiveness.

### 4.2. Offspring network average degree

Unweighted network average degree describes the average number of contacts with different siblings in the brood [98–100]. Here, we used network average degree as a measure of sociability, which we expected to be higher in broods from females breeding in areas with a simulated high social density. However, the experimental treatment did not significantly affect network average degree. It is possible that the brood sizes we analysed were not large enough to obtain meaningful networks, which could preclude finding a significant effect. However, our results do reveal a significant association with testosterone (see below). Additionally, the lack of a treatment effect could be due to the fact that we stopped the social cue manipulation prior to hatching, while network average degree could result from an interplay between the pre- and post-natal environments (e.g. [34]). Females may adjust offspring phenotype prior to hatching via maternal effects, but it is also possible that maternal behaviour during parent–offspring interactions after hatching impacts brood cohesiveness, as shown in great tits, albeit in a different context [11]. This remains speculative at the moment.

We found a negative relationship between yolk testosterone and network average degree. The lower yolk testosterone concentrations observed in the simulated high social density imply that these broods were primed to develop a more interactive and social phenotype. This aligns with our prediction that nestlings born in socially dense environments should be maternally programmed to be more sociable. Higher yolk testosterone is often associated with more aggressive and competitive phenotypes in birds [64,65]. In humans, higher prenatal testosterone has been found to be associated with a higher sociability and a larger social group size [101], but little is known about this relationship in other taxa. Nevertheless, personality traits are closely intertwined and trade-offs between them inevitably arise [102]. Therefore, our results could be explained by a negative correlation between sociability and aggressiveness, mechanistically mediated by prenatal yolk testosterone. In line with this, previous research has found that aggressive individuals are less tolerant to the presence of conspecifics [94,95].

### 4.3. Parent–offspring interactions

Even though competition was not enhanced for the adults, simulated higher social densities might have been perceived as an environment where access to food would be more limited and parental care more constrained. Another explanation for the observed pattern of yolk testosterone allocation could be that clutches with lower yolk testosterone result in less demanding offspring, given the positive effects of testosterone on begging [103–105] and metabolic rate [106]. Producing offspring with reduced demand could therefore be adaptive in a high social density scenario. However, yolk testosterone did not influence nestling begging intensity. While it is possible that the manipulation of the maternal environment had smaller effects on begging compared with direct yolk hormone manipulations [67], previous research has shown mixed evidence regarding the relationship between maternal yolk testosterone and begging behaviour (see review by [107]). Additionally, it has been suggested that female birds use yolk hormones to increase male parental investment by modifying offspring begging behaviour [108]. We found that nestlings from experimentally treated nests begged more intensively to their fathers than to their mothers. This suggests that, even though maternal yolk testosterone does not mediate the development of more or less demanding offspring, other components in the yolk could play a role in resolving sexual conflict in this species.

There were no differences in feeding frequency between parents related to the manipulation, and both sexes tended to feed nestlings more often when yolk testosterone concentrations were higher, though this relationship was not significant. In these broods, females also tended to spend less time in the nest after feeding. Females who spend more time foraging and feeding the nestlings cannot dedicate as much time to nest attendance [109]. Thus, the positive tendency of yolk testosterone with feeding frequency and the negative one with time spent in the nest could be explained by the trade-off between these two behaviours. This is further supported by the negative effect of brood size on time spent in the nest, as larger broods require females to invest more time in foraging [110]. However, these trends were too subtle to result in significant differences in nestling body mass.

## 5. Conclusions

A simulated high social density resulted in a reduced allocation of maternal testosterone by females. This suggests that, in the absence of competition, lower testosterone levels could be adaptive, as they were associated with the development of a more sociable phenotype. This highlights the importance of hormone-mediated maternal effects as an intergenerational link between prenatal social conditions and the acquisition of social traits. Further studies are needed to determine whether these effects are lasting, whether offspring benefit from them in high social density environments, or how they could affect territory choices, for example, through phenotype-environment matching.

## Data Availability

All the datasets supporting this article have been uploaded to the Dryad Repository [111]. Supplementary material is available online [112].
